# Increased Risk of Benign Prostate Hyperplasia in Sleep Apnea Patients: A Nationwide Population-Based Study

**DOI:** 10.1371/journal.pone.0093081

**Published:** 2014-03-25

**Authors:** Ping-Song Chou, Wei-Chiao Chang, Wei-Po Chou, Mu-En Liu, Chiou-Lian Lai, Ching-Kuan Liu, Yan-Chiou Ku, Shih-Jen Tsai, Yii-Her Chou, Wei-Pin Chang

**Affiliations:** 1 Department of Neurology, Kaohsiung Medical University Hospital, Kaohsiung, Taiwan; 2 Department of Clinical Pharmacy, School of Pharmacy, Taipei Medical University, Taipei, Taiwan; 3 Department of Psychiatry, Kaohsiung Medical University Hospital, Kaohsiung, Taiwan; 4 Department of Psychiatry, Kaohsiung Veterans General Hospital, Kaohsiung, Taiwan; 5 Department of and Master’s Program in Neurology, Faculty of Medicine, College of Medicine, Kaohsiung Medical University, Kaohsiung, Taiwan; 6 Nursing Department, Kaohsiung Veterans General Hospital, Kaohsiung, Taiwan; 7 Department of Psychiatry, Taipei Veterans General Hospital, Taipei, Taiwan; 8 Department of Urology, Faculty of Medicine, College of Medicine, Kaohsiung Medical University, Kaohsiung, Taiwan; 9 Department of Urology, Kaohsiung Medical University Hospital, Kaohsiung, Taiwan; 10 Department of Healthcare Management, Yuanpei University, Hsinchu, Taiwan; University of British Columbia, Canada

## Abstract

**Background:**

Sleep apnea (SA) is a common sleep disorder characterized by chronic intermittent hypoxia (IH). Chronic IH induces systemic inflammatory processes, which can cause tissue damage and contribute to prostatic enlargement. The purpose of this study was to evaluate the association between benign prostate hyperplasia (BPH) and SA in a Taiwanese population.

**Methods:**

The study population was identified from Taiwan’s National Health Insurance Research Database (NHIRD) and contained 202 SA patients and 1010 control patients. The study cohort consisted of men aged ≥30 years who were newly diagnosed with SA between January 1997 and December 2005. Each patient was monitored for 5 years from the index date for the development of BPH. A Cox regression analysis was used to calculate the hazard ratios (HRs) for BPH in the SA and control patients.

**Results:**

During the 5-year follow-up, 18 SA patients (8.9%) and 32 non-SA control patients (3.2%) developed BPH. The adjusted HR for BPH was 2.35-fold higher in the patients with SA than in the control patients (95% confidence interval (CI) 1.28–4.29, P<.01). We further divided the SA patients into 4 age groups. After adjusting for potential confounding factors, the highest adjusted HR for BPH in the SA patients compared with the control patients was 5.59 (95% CI = 2.19–14.31, P<.001) in the patients aged between 51 and 65 years.

**Conclusion:**

Our study results indicate that patients with SA are associated with increased longitudinal risk of BPH development, and that the effects of SA on BPH development are age-dependent.

## Introduction

Sleep apnea (SA) is a common sleep disorder characterized by multiple cessations of breath during sleep, which cause intermittent hypoxia (IH) and sleep fragmentation. Each cessation of breath, referred to as an apnea, can last from 10 seconds to minutes. SA can be mild, moderate, or severe, according to the number of apneas per hour [1]. Increasing evidences indicate that SA is a risk factor for diabetes, stroke, cardiovascular mortality and morbidity [2]–[4]. IH is one of the major pathological conditions to affect SA patients, and chronic IH can activate inflammatory pathways [5]. Studies have indentified elevated systemic pro-inflammatory cytokines and inflammatory markers in SA patients [6], [7]. Abnormal inflammatory status in SA could be a key pathogenic factor in cardiovascular events [6], [7]. Therefore, SA is considered a systemic inflammatory disease [8].

Benign prostate hyperplasia (BPH) is the most frequent benign neoplasm in aging men and one of the most common chronic conditions in the male population, with a histological prevalence at autopsy of 50% in men aged 50–60 years and 90% in men aged over 80 years [9]. Although the pathogenesis of BPH is not yet fully understood, recent evidence indicates the role of chronic inflammation in BPH [10]. Robert et al identified the wide distribution of inflammatory elements (ie, reactive oxygen species (ROS), lymphocytes, macrophages, neutrophils, and cytokines) in surgically treated BPH tissues [11]. Such chronic inflammatory conditions may contribute to tissue injury, activate cytokines release, increase the concentration of growth factors, and subsequently cause the development of BPH [12].

SA-mediated chronic IH induces systemic inflammatory processes [5], which can cause tissue damage and continuous wound healing, thus contributing to prostatic enlargement [10]. This mechanism suggests that long-term SA may lead to BPH development through chronic IH. However, according to our review, no study has demonstrated increased incidence of prostatic neoplasm in patients with long-term SA. In this study, we examined the association between BPH and SA by using a nationwide population-based data set in Taiwan. We also evaluated the age-dependent effects of SA on the development of BPH.

## Materials and Methods

### Database

The National Health Insurance (NHI) program is a compulsory health insurance program in Taiwan. The program provides comprehensive coverage for all resident medical care nationwide. Approximately 99% of Taiwan’s population was enrolled in the program by 2009. All claims data are collected in the National Health Insurance Research Database (NHIRD) at the National Health Research Institutes (NHRI). This study used a data set from the Longitudinal Health Insurance Database 2005 (LHID2005), which is also managed by the NHRI. The data consist of ambulatory care records, inpatient care records, and the registration files of 1,000,000 randomly sampled insurants from the total number of NHRI beneficiaries from 1996 to 2010. The NHRI states that no statistical differences in age, sex, and health care costs exist between the sampled group and all enrollees.

In the LHID2005 database, each patient’s original identification number was encrypted for privacy in the cohort data set. The encryption procedure was consistent between datasets so that all claims belonging to each patient could be linked. To our knowledge, the LHID2005 data set is one of the largest nationwide population-based databases worldwide and numerous scientific studies have been published using its data [13], [14].

### Study Population

The study cohort consisted of 202 patients aged ≥30 years who were newly diagnosed with SA (International Classification of Diseases, Ninth Revision, Clinical Modification (ICD-9-CM) codes 327.23, 780.51, 780.53, 780.57) between January 1997 and December 2005. To ensure the accuracy of data, the included patients must have received a polysomnogram (NHI code 17008B). All ICD-9-CM codes were assigned by an otolaryngologist, a pulmonologist, or a neurologist. Between January 1997 and December 2005, the initial SA diagnosis date was set as the index date for each patient. Patients with SA before or after the enrollment period and patients who had previously been diagnosed with BPH (ICD-9-CM code 600.X) were excluded from analyses.

The inclusion criteria for BPH included the diagnosis of BPH by an urologist through an ultrasound examination of the prostate (NHI code 19005B or 19017C) and medical treatment for BPH, including Tamsulosin (ATC codes B024403100, B025413100), Doxazosin (ATC codes B021643100, B021641100, B023711100), and Terazosin (ATC code B017664100).

The patients were monitored for 5 years from the index date to determine the development of BPH. Several covariables, such as hypertension (ICD-9-CM codes 401.X–405.X), diabetes mellitus (ICD-9-CM code 250.X), and hyperlipidemia (ICD-9-CM code 272.X), were included in the analytical model to examine the relationship between SA and particular comorbidities.

The comparison cohort was randomly sampled from the remaining patients of the LHID2005 data set. Patients were excluded if they were diagnosed with or had a history of SA or BPH. The final comparison cohort was randomly selected from the LHID2005 data set at a control-to-case ratio of 5∶1, matched according to age and index year.

### Level of Urbanization

The criteria published by the Taiwanese NHRI on urbanization levels in Taiwan were adopted, which include 7 strata, with Level 1 denoting the most urbanized communities and Level 7 denoting the least urbanized communities. All 359 communities in Taiwan were stratified into the 7 levels. The standards for classification included population density (people/km2), proportion of people with a college education or above, proportion of people aged >65 years, proportion of agricultural workers, and number of physicians per 100 000 people. However, because of the small numbers of SA cases in Levels 4, 5, 6, and 7, these 4 levels were combined into a single group, referred to as Level 4.

### Ethical Approval

This study was conducted in accordance with the Helsinki Declaration. A formal waiver was received from the Institutional Review Board (IRB) of Kaohsiung Medical University Hospital (KMUH-IRB-EXEMPT-20130065).

### Statistical Analysis

All data processing and statistical analyses were performed using Statistical Package for the Social Sciences (SPSS) software, Version 18 (SPSS, Inc). Pearson chi-square tests were used to compare differences in geographic location, monthly income, and the urbanization level of patients’ residences between the study group and control group. A stratified Cox proportional hazards regression analysis (stratified by age group and index year) was performed to examine the risk of BPH development during the 5-year follow-up in patients with and without SA. All patients were monitored from the index date until BPH presentation or the end of the 5-year follow-up. Hazard ratios (HRs) and 95% confidence intervals (CIs) were calculated to indicate the risk of BPH. A 2-sided P value <.05 was considered statistically significant.

## Results

**Figure 1:**
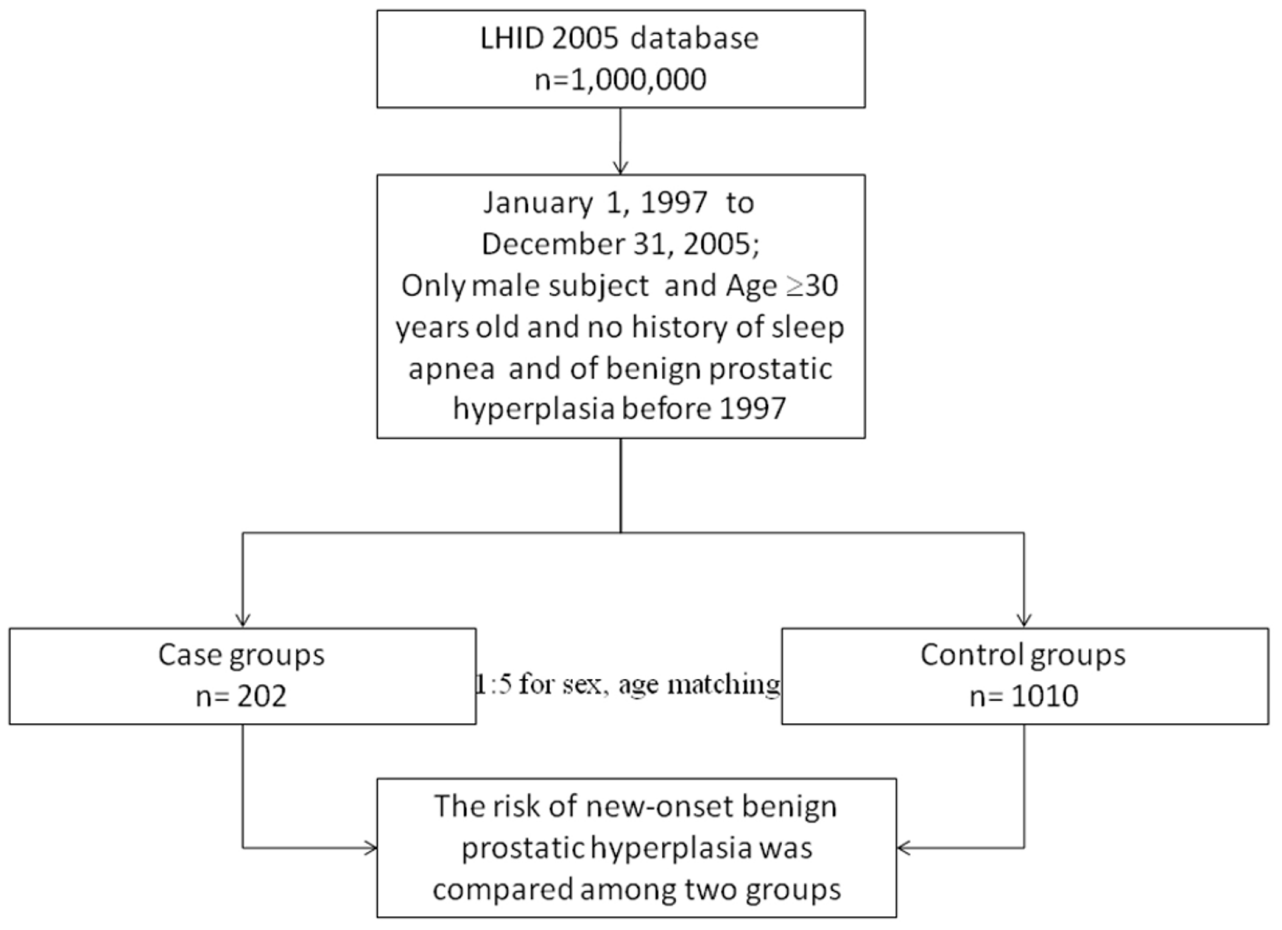
The research design flow chart of the present study.

**Figure 2:**
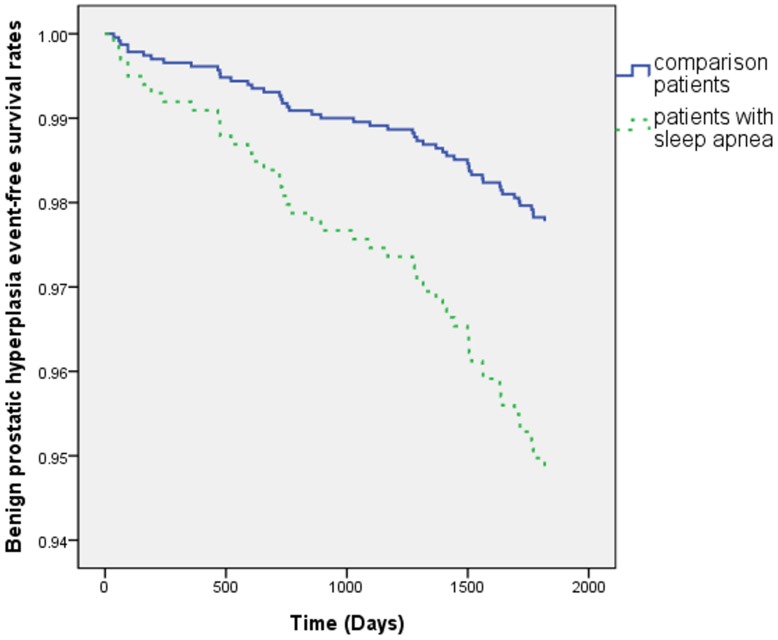
Benign prostate hyperplasia–free survival rates for patients with sleep apnea and control groups from 1997 to 2005.

Figure 1 shows the research design flowchart. Of the patients diagnosed with SA, 202 patients fulfilled the inclusion criteria. The comparison cohort contained 1010 patients. Table 1 lists the distributions of the sociodemographic characteristics and the comorbid medical disorders in the SA and comparison groups. The SA patients were significantly more likely to experience hypertension (P<.001), hyperlipidemia (P<.001), and diabetes (P<.001) than the control patients were. During the 5-year follow-up, 18 SA patients (8.9% of the study cohort) and 32 non-SA patients (3.2% of the comparison cohort) developed BPH. The Cox regression analysis demonstrated that the crude HR for BPH was 2.91-fold higher in the patients with SA than in the comparison cohort (95% CI = 1.63–5.18, P<.001). The HR remained significant after adjusting for potential confounders (hypertension, hyperlipidemia, diabetes, geographic region, and monthly income variables). As shown in Table 2, the adjusted HR was 2.35-fold higher in the patients with SA than in the comparison cohort (95% CI = 1.28–4.29, P<.01). BPH development correlated with SA in our study patients. The Cox proportional hazards regression analysis indicated that the SA patients were associated with significantly lower 5-year BPH event-free survival rates in comparison with the control patients (P<.01) (Fig. 2).

**Table 1:** Demographic characteristics for the selected patients, stratified by presence/absence of sleep apnea from 1997 to 2005 (n = 1212).

	Patients with Sleep apnea (n = 202)		Patients without Sleep apnea (n = 1010)		P value
	n	%	N	%	
**AGE(Years)**					1
≦35	29	14.4	145	14.4	
36–50	119	58.9	595	58.9	
51–65	45	22.3	225	22.3	
≧66	9	4.5	45	4.5	
**Follow-up, year, mean (SD)**					0.02
	4.78	0.86	4.93	0.48	
**Urbanization level**					0.05
1(most urbanized)	87	43.1	336	33.3	
2	50	24.8	271	26.8	
3	33	16.3	185	18.3	
4(least urbanized)	32	15.8	218	21.6	
**Monthly income**					0.003
0	16	7.9	91	9.0	
NT$ 1–15840	19	9.4	142	14.1	
NT$ 15841–25000	53	26.2	348	34.5	
≧25001	114	56.4	429	42.5	
**Geographic region**					0.003
North	127	62.9	519	51.4	
Central	47	23.3	261	25.8	
South	28	13.9	195	19.3	
Eastern	0	0	35	3.5	
**Hypertension**					<0.001
Yes	115	56.9	320	31.7	
No	87	43.1	690	68.3	
**Hyperlipidemia**					<0.001
Yes	101	50.0	298	29.5	
No	101	50.0	712	70.5	
**Diabetes**					<0.001
Yes	59	29.2	172	17.0	
No	143	70.8	838	83.0	

**Table 2:** Hazard ratios (HRs) of benign prostatic hyperplasia among sleep apnea patients during the 5-year follow-up period from the index ambulatory visits or inpatient care from 1997 to 2005.

	Total		Patients with sleep apnea		Patients without sleep apnea	
Development of benign prostatic hyperplasia	NO.	(%)	NO.	(%)	NO.	(%)
5-year follow-up period						
Yes	50	4.1	18	8.9	32	3.2
No	1162	95.9	184	91.1	978	96.8
Crude HR (95% CI)			2.91 (1.63–5.18)***		1	
Adjusted HR (95% CI)			2.35 (1.28–4.29)**		1	

Total sample number = 1212.

Both crude and adjusted HRs were calculated by Cox proportional hazard regressions, and stratified by age and sex.

Adjustments were made for patients’ Age, Geographic region, Monthly income, Hypertension, Hyperlipidemia, and Diabetes.

** Indicates p<0.01;

*** Indicates p<0.001.

To evaluate whether SA is an age-dependent risk factor for BPH, we divided the SA patients into 4 age groups: ≤35 years, 36–50 years, 51–65 years, and >66 years. After adjusting for potential confounding factors, the highest adjusted HR for BPH in the patients with SA in comparison with the control patients was 5.59 (95% CI = 2.19–14.31, P<.001) in the patients aged 51–65 years (Table 3).

**Table 3:** Hazard ratios for Benign prostatic hyperplasia among patients with Sleep apnea and the comparison cohort by age group.

	Age Group							
Development ofbenign prostatichyperplasia	≦35		36–50		51–65		≧66 or older	
	study group	comparison	study group	comparison	study group	comparison	study group	comparison
	n (%)	n (%)	n (%)	n (%)	n (%)	n (%)	n (%)	n (%)
Yes	0	0	8 (6.7)	13 (2.2)	10 (22.2)	12 (5.3)	0	7 (15.6)
Crude HR (95% CI)	–	1	3.17 (1.31–7.64)*	1	4.66 (2.01–10.78)***	1	–	1
Adjusted HR (95%CI)	–	1	2.06 (0.80–5.34)	1	5.59 (2.19–14.31)***	1	**–**	1

Adjustments were made for patients’ Age, Geographic region, Monthly income, Hypertension, Hyperlipidemia, and Diabetes.

*** Indicates p<0.001.

## Discussion

According to our research, our study is the first longitudinal large-scale nationwide study to demonstrate increased risk of BPH in patients recently diagnosed with SA over a 5-year period. Our principal study finding was a 2.35-fold higher HR for BPH in the patients with SA than in the control group, after stratifying for patient age, sex, and year of index date. This finding supported our hypothesis that SA patients are associated with increased risk of BPH development. Our study results confirmed that the prevalence of hypertension, hyperlipidemia, and diabetes is higher in SA patients than in control patients, and also indicated lower income in the SA patients than in control patients. These findings are consistent with previously reported results [15].

Several possible factors could explain our findings. First, IH is a trigger for systemic inflammation and oxidative stress, which might promote development of BPH [16], [17]. Multiple inflammatory markers and mediators, including tumor necrosis factor- alpha (TNF-α), interleukin (IL)-6, IL-8, C-reactive protein, transcription factor and adhesion molecules are all elevated in patients with SA [18], [19]. These inflammatory mediators can induce the growth of prostatic epithelial and stromal cells [10], [16], [20], [21]. IH can also promote the accumulation of the transcriptional activator hypoxia-inducible factor 1α (HIF-1α) [22], which is a key factor in the progression of prostate hyperplasia and a potential therapeutic target in BPH [23]. Second, SA might accelerate atherosclerosis by increasing lipid loading in macrophages, lipid peroxidation, and endothelial dysfunction. [18]. The atherosclerotic condition can reduce blood supply to the prostate and has been identified as a risk factor for BPH development [24], [25]. Third, SA is involved in insulin resistance [26], [27], which is a well-established risk factor for BPH [28]–[30]. Fourth, SA and BPH are complex diseases with genetic and environmental factors involved in their development. Previous studies have indicated that TNF-α gene promoter polymorphism is associated with an increased risk of obstructive SA [31] and observed the association between TNF-α gene promoter polymorphism and risk of BPH [32]. These findings indicate a shared genetic background between SA and BPH.

Our study results also indicated the age-associated effects of SA on BPH development in male patients aged <65 years. These effects were most evident in men aged between 51 and 65 years (Table 3). However, the mechanism underlying these age-associated effects remains unclear.

By using magnetic resonance imaging (MRI), Williams et al identified age-associated increases in prostate growth rate, observing that prostate growth rate peaks in men aged 56–65 years and then declines rapidly in older men [33]. Schenk et al suggested that the prostate is less sensitive to inflammatory and oxidative stress in men aged >65 years than in younger men [34]. Therefore, SA patients aged <65 years might be more susceptible to BPH development than those aged >65 years.

The findings from our nationwide population-based cohort study support the association between SA and BPH. Studies on SA treatment have indicated that continuous positive airway pressure (CPAP) therapy can effectively reduce SA nocturnal hypoxia and its associated co-morbidity [35]. If overnight hypoxia in SA patients plays a major role in increased risk of BPH during follow-up, treatment such as CPAP might decrease the risk of BPH in SA patients.

Our study strengths include its matched control cohort study design, which considered several variables to minimize the effects of potential confounding factors. Because the NHI program in Taiwan is a single-payer, mandatory health insurance program with affordable payments, the majority of events can be traced and referral bias minimized.

However, some study limitations exist, which might have influenced our findings. First, NHIRD claims data are available only from 1997 onward; therefore, it was not possible to determine the exact duration of SA and the onset of BPH in each patient. Second, the NHIRD does not contain data on education, family history, tobacco smoking status, body mass index, or alcohol consumption, which could all represent confounding factors. Third, the NHIRD does not contain data on patients’ International Prostate Symptom Score (IPSS), prostate volume, or apnea-hypopnea index. Therefore, the severity of BPH and SA cannot be determined using the data in the NHIRD. Fourth, men with SA are more likely to experience nocturia than men without SA and young men with SA are more likely to experience nocturia than elderly men with SA are [36]. Therefore, the confounding factor of nocturia in young men might have influenced our results. Fifth, we diagnosed BPH though ultrasound examination of the prostate. However, periurethral BPH can cause lower urinary tract symptoms with relatively small prostate volume. Therefore, we might have underestimated the number of BPH patients in this study. In addition, the sample of SA patients was small, which might have caused inadequate power in our statistical analyses. Finally, the treatment status of and compliance with CPAP, a major treatment in SA patients, could not be determined from the registry. Future studies that consider these indices for the evaluation of the association between SA and BPH are warranted.

## Conclusion

Our study results support that SA patients are associated with higher longitudinal risk of BPH development in comparison with non-SA patients, particularly in populations aged 51–65 years. Further investigation is required to elucidate the pathological mechanisms underlying these 2 diseases.
